# Endothelin potentiates TRPV1 via ET_A _receptor-mediated activation of protein kinase C

**DOI:** 10.1186/1744-8069-3-35

**Published:** 2007-11-14

**Authors:** Tim D Plant, Christian Zöllner, Frauke Kepura, Shaaban S Mousa, Jenny Eichhorst, Michael Schaefer, Jens Furkert, Christoph Stein, Alexander Oksche

**Affiliations:** 1 Institut für Pharmakologie und Toxikologie, FB-Medizin, Philipps-Universität Marburg, Karl-von-Frisch-Str. 1, 35032 Marburg, Germany; 2 Institut für Pharmakologie, Charité-Universitätsmedizin Berlin, Campus Benjamin Franklin, Thielallee 67-73, 14195 Berlin, Germany; 3 Klinik für Anästhesiologie und operative Intensivmedizin, Charité-Universitätsmedizin Berlin, Campus Benjamin-Franklin, Hindenburgdamm 30, 12200 Berlin, Germany; 4 Leibniz-Institut für Molekulare Pharmakologie, Robert-Rössle-Str. 10, 13125 Berlin, Germany

## Abstract

**Background:**

Endothelin-1 (ET-1) both stimulates nociceptors and sensitizes them to noxious stimuli, an effect probably mediated by the ET_A _receptor (ET_A_R) expressed in sensory neurons. The cellular mechanisms of this ET-1-mediated effect are only poorly understood. TRPV1, the heat-, pH- and capsaicin-sensitive cation channel already known to be modulated by a number of cellular mediators released in response to noxious stimuli and during inflammation, is a potential target for the action of ET-1.

**Results:**

We studied the effects of ET-1 on TRPV1 in sensory neurons from the dorsal root ganglion (DRG) and in HEK293 cells coexpressing TRPV1 and the ET_A_R. Specific ^125^I-ET-1 binding sites (817 ± 92 fmol/mg) were detected in membrane preparations of DRG with an ET_A_R/ET_B_R ratio of 60:40. In an immunofluorescence analysis, coexpression of TRPV1 and the ET_A_R was found in a subpopulation of primary sensory neurons. ET-1 strongly potentiated capsaicin-induced TRPV1 currents in some neurons, and in HEK293 cells co-expressing TRPV1 and the ET_A_R. Weaker potentiation was observed in HEK293 cells coexpressing TRPV1 and the ET_B_R. ET_A_R activation also increased responses to low pH and heat. In HEK293 cells, strong potentiation of TRPV1 like that induced by ET-1 via the ET_A_R could be induced by PKC activation, but not with activators of the adenylyl cyclase or the PKA pathway. Furthermore, inhibition of PKC with bisindolylmaleimide X (BIM X) or mutation of the PKC phosphorylation site S800 completely prevented ET_A_R-mediated potentiation.

**Conclusion:**

We conclude that ET-1 potentiates TRPV1 by a PKC-dependent mechanism and that this could play a major role in the algogenic and hyperalgesic effects of ET-1 described in previous studies.

## Background

Endothelin is one of many local mediators that are important in pain generation and the modulation of nociceptor responsiveness to painful stimuli. The endothelins, ET-1, ET-2 and ET-3, are vasoactive peptides, originally cloned from endothelial cells [1], but also produced by other cell types, including some tumor cells [2-5]. Endothelins act on ET_A _and ET_B _receptors (ET_A_Rs and ET_B_Rs) [6,7], both G protein-coupled receptors that can activate multiple G protein types and influence various signaling pathways [8].

ET-1 injection excites nociceptors [9,10] and induces nocifensive behaviour in animals [11-13], and severe pain and tactile allodynia in humans [14]. ET receptor antagonists have been reported to reduce neuropathic and inflammatory pain, and pain in patients with metastatic prostate cancer (see [15,16] for reviews). Given the number of reports on the involvement of ET-1 in nociception, relatively little is known about the signaling cascade and effectors that lead to the nociceptive responses to ET-1 in primary sensory neurons.

Activation of the ET_A_R, which is expressed in sensory neurons [17], results in small increases in [Ca^2+^]_i _in a sensory neuron-derived cell line [18] and DRG neurons [19], and in a protein kinase C(PKC)-ε-mediated potentiation of Ca^2+ ^responses to capsaicin [19]. The increased responsiveness of sensory neurons may result from an ET_A_R-mediated lowering of the threshold for activation of tetrodotoxin (TTX)-insensitive Na^+ ^channels [20], but may involve other effectors. One possibility is that ET-1 affects other channels like the nonselective cation channel TRPV1, an integrator of a number of noxious stimuli, including heat (> 42°C), capsaicin, endocannabinoids and H^+ ^[21], which is essential for thermal hyperalgesia in inflammation [22,23]. TRPV1 activation results in depolarization and excitation of sensory neurons. In a preliminary conference report we showed that activation of the ET_A_R potentiated TRPV1 responses to capsaicin in HEK 293 cells [24]. A number of modulators sensitize nociceptors by potentiating TRPV1 responses [25-30]. Possible mechanisms involved in potentiation are phosphorylation *via *PKC-ε [31] and protein kinase A (PKA) [32,33], disinhibition of TRPV1 by hydrolysis of phosphatidylinositol bisphosphate (PIP_2_) [28], or modulation via phophatidylinositol-3-kinase and extracellular signal-related kinases 1/2 [34].

In this study, we investigated ET receptor expression in DRG and, using the patch clamp technique, the effects of ET-1 on responses to capsaicin in DRG neurons. A subpopulation of neurons responded to ET-1 with a potentiation of the capsaicin-mediated responses. To investigate the signaling pathways involved in potentiation, we studied the effects of ET-1 in HEK293 cells coexpressing the ET_A_R and TRPV1.

## Results

### Endothelin receptors in dorsal root ganglion neurons

The expression of endothelin receptor subtypes in the rat lumbar DRG was analyzed in binding experiments using ^125^I-ET-1 as the radioligand. Saturation binding analysis of membranes derived from isolated lumbar DRG (L4 – L5) revealed a maximal binding capacity of 817 ± 92 fmol/mg (mean ± SD of three measurements performed in duplicate). When the ET_A_R-selective antagonist BQ123 or the ET_B_R-selective agonist IRL1620 were used as competing ligands (to determine the amount of ET_A_R or ET_B_R expression), binding capacities of 503 ± 115 and 313 ± 113 fmol/mg protein were obtained, respectively. Thus, both ET_A_Rs and ET_B_Rs are expressed in the rat DRG with an expression ratio of 60:40.

**Figure 1:**
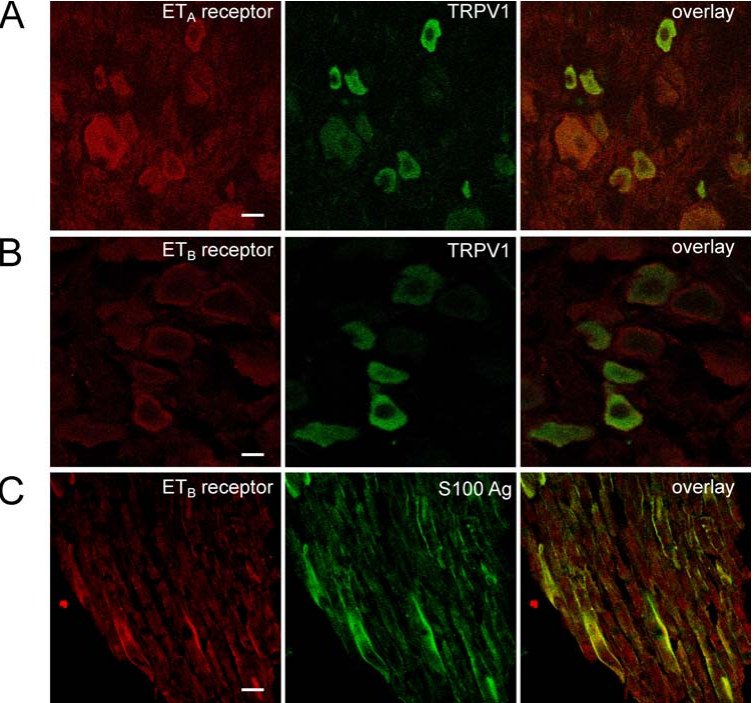
**Expression of ET_A_Rs and ET_B_Rs in rat dorsal root ganglion**. Sections of lumbar rat dorsal root ganglion were analysed for the expression of ET_A_Rs (A) or ET_B_Rs (B, C) using affinity-purified antibodies. For the identification of TRPV1-postive neurons co-staining with a TRPV1 antibody (A, B) was performed. Expression of ET_B_Rs in glial cells was demonstrated by co-staining of the S100 antigen (C). Bars: 20 μm.

In an immunofluorescence analysis, we further studied the distribution of ET_A_Rs and ET_B_Rs in tissue sections of lumbar DRG. To probe whether ET_A_Rs and ET_B_Rs are expressed in small sensory neurons that express TRPV1, we performed double staining experiments using affinity-purified antibodies against ET_A_Rs or ET_B_Rs in conjunction with antibodies against TRPV1. The data show that ET_A_Rs are widely expressed in small and medium-to-large diameter neurons, and, in particular, in TRPV1-expressing small sensory neurons (Fig. 1A). Controls with pre-immune serum showed a weak uniform staining of all neurons (not shown). Coexpression of the ET_A_R with TRPV1 was found in 31 of 66 TRPV1-positive DRGs (47%). ET_B_R expression in DRGs was, if present, relatively weak (Fig. 1B), and not easy to distinguish from unspecific staining (not shown). The staining with the ET_B_R antibody showed a different pattern from that of the ET_A_R and TRPV1 antibodies and surrounded the perikarya, possibly reflecting expression in satellite cells. More prominent staining with the ET_B_R antibody was observed when analyzing the axonal extensions of DRG, where the cells stained by the ET_B _antibody were costained by antibodies directed against the S100 antigen (Fig. 1C), indicating that they are glia. In contrast, for the ET_A_R no expression was found in glial cells (data not shown). Thus, ET_A_R are found in sensory neurons, including TRPV1-positive small diameter neurons, whereas ET_B_Rs are mainly found in glial cells. Our data on ETR expression in sensory neurons are consistent with previous reports [17,35], which also demonstrated ET_A_R expression in neurons, frequently in small neurons together with calcitonin gene-related peptide [17], a marker of a subpopulation of C fibers. These studies also demonstrated ET_B_R expression in DRG satellite cells and nonmyelinating Schwann cells, but not in DRGs.

### Effects of ET-1 in isolated sensory neurons

**Figure 2:**
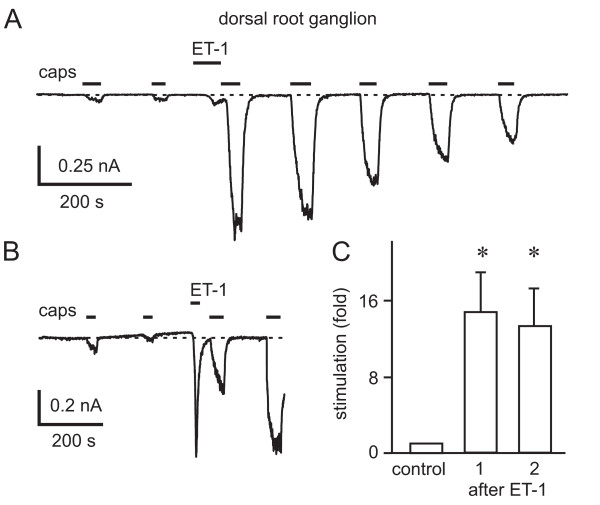
**Potentiation of capsaicin-activated currents in dorsal root ganglion neurons by ET-1**. A, Recording of current at -60 mV showing responses to repetitive applications of capsaicin (10 nM) in a Ca^2+^-free extracellular solution. Application of ET-1 (100 nM) at the time indicated by the bar resulted in a small increase in current during the ET-1 application and a large potentiation of the capsaicin-activated currents. B, Results of a similar experiment to that in A which shows a larger current response to ET-1 application. C, Bar graph summarising the potentiation of capsaicin responses by ET-1 (100 nM) in ET-1-responsive rat DRG neurons. *Control *is the normalized capsaicin response before ET-1 application, *after ET-1 *the first (*n *= 9, *p *= 0.0066) and second (*n *= 8, *p *= 0.017) normalized responses after ET-1 application.

A low concentration of capsaicin (10 nM) evoked currents through TRPV1 in a subpopulation of DRG neurons. In the Ca^2+^-free extracellular solutions used to minimise the Ca^2+^-dependent component of desensitization, repetitive applications of capsaicin activated currents which were either stable or which declined slowly. In 9/30 capsaicin-sensitive neurons (30%), application of ET-1 (100 nM) for 30 or 60 s prior to the application of 10 nM capsaicin resulted in a large increase in the capsaicin-induced current (Fig. 2A). On average, currents were increased 14.7 ± 4.1-fold (mean ± SEM, *n *= 8) when the first capsaicin-elicited response after ET-1 treatment was compared to that before ET-1 addition. The potentiation of the capsaicin responses by a single application of ET-1 was transient; the amplitude of the capsaicin-activated current declined to different degrees in response to repetitive stimulation in the six neurons in which three or more capsaicin responses could be elicited after ET-1 application. However, as shown in Figure 2A, potentiation often persisted for several minutes. In 5/9 neurons that responded to ET-1 with a potentiation of capsaicin-induced TRPV1 activation, ET-1 alone also transiently activated an inward current with a wide range of amplitudes (between -2.0 and -69.3 pA/pF, mean: -25.5 ± 12.3 pA/pF, *n *= 5) at -60 mV (Fig. 2B). Activation and decay of the ET-1-activated current was rapid. The cells that responded to ET-1 with a potentiation of capsaicin-activated currents could not be distinguished from the non-responsive cells on the basis of their size. Their capacitances, indicative of the cell surface area, were 35.3 ± 5.9 pF (*n *= 8) and 39.1 ± 3.7 pF (*n *= 21), respectively. In 13 of the 21 cells that did not respond to ET-1, we subsequently tested for the ability of bradykinin (1 μM) to potentiate capsaicin (10 nM)-activated TRPV1 currents. In 6/13 cells, bradykinin application resulted in a potentiation of capsaicin-activated currents through TRPV1 (16.5 ± 7.6-fold, *n *= 6), which closely resembled that induced by ET-1 (data not shown). It was notable that responses of DRGs to ET-1 were mainly observed on the day of preparation, or on the day after preparation. Neurons cultured for longer rarely responded to ET-1, but did respond to bradykinin, suggesting that in cultured DRGs the expression of ET receptor subtypes might be down regulated or that there is an impairment in the efficiency of coupling to downstream signaling cascades. Loss of receptors during culture may also explain the lower percentage of cultured capsaicin-sensitive neurons that respond to ET-1, compared to the percentage of DRGs coexpressing TRPV1 and ET_A_R in sections of ganglia.

### Effects of ET-1 on HEK293 cells transiently transfected with TRPV1 and the ET_A _or ET_B _receptor

**Figure 3:**
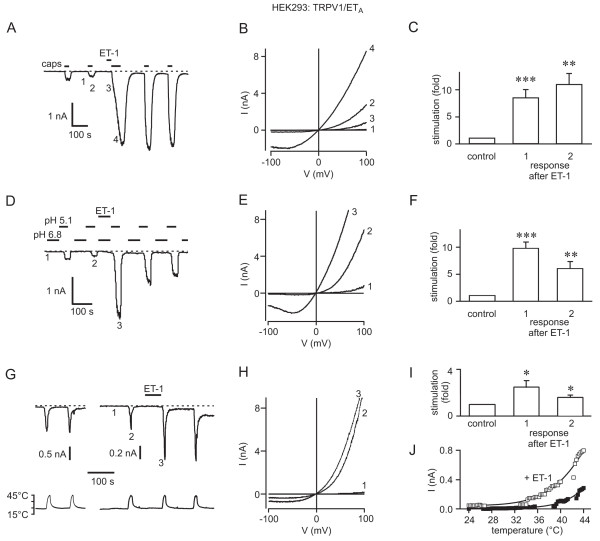
**Potentiation of TRPV1 currents by ET-1 in HEK293 cells cotransfected with TRPV1 and the ET_A_R**. A – C, Potentiation of capsaicin-activated currents by ET-1. A, Recording of current at -60 mV showing the potentiation of responses to capsaicin (10 nM) by the application of ET-1 (100 nM). B, IV-relationships recorded at the times indicated by the numbers 1 – 4 in A. C, Bar graph summarizing the potentiation of TRPV1 responses to capsaicin by ET_A_R activation in HEK293 cells. The bars show the normalized control response prior to ET-1 application and the normalized first (*n *= 10, *p *= 0.0009) and second (*n *= 9, *p *= 0.0012) responses after ET-1 application. D – F, Potentiation of H^+^-activated currents by ET-1. D, ET-1 increased the amplitude of TRPV1 responses to pH 5.1. Currents were recorded at -60 mV and applications of pH 5.1 preceded by a solution of pH 6.8 to prevent the activation of endogenous ASICs. E, IV-relationships recorded at the times indicated by numbers in D. F, Bar graph summarizing the potentiation of the TRPV1 response to pH 5.1 by ET-1, and showing the normalized response before ET-1, and the first (*n *= 12, *p *< 0.0001) and second (*n *= 9, *p *= 0.0069) responses after ET-1. G – J, Potentiation of heat-activated currents through TRPV1 by ET-1. G, Current recordings at -60 mV (*upper traces*) showing consecutive responses of a control cell to heating to 44°C (*left*), and responses from a cell treated with ET-1 after the first response to heat (*right*). The *lower traces *show the temperature recorded in the chamber close to the cell. H, I-V relationships recorded at the times indicated by the numbers in G. I, Bar graph summarizing the potentiation of the normalized first (*n *= 10, *p *= 0.0252) and second (*n *= 7, *p *= 0.0233) responses to heat after ET-1. J, Plot of the current-temperature relationships for the control response (*filled squares*) and the second response after ET-1 (*open squares*) from the experiment in G.

Owing to the small fraction of neurons that responded to ET-1, the regulation of TRPV1 by ET-1 was analysed in HEK293 cells transiently co-transfected with plasmids encoding TRPV1-YFP and the ET_A_R or ET_B_R. HEK293 cells co-expressing the ET_A_R and TRPV1 responded to 10 nM capsaicin with currents that displayed the characteristic outwardly-rectifying IV-relation of TRPV1 (Fig. 3A, B). ET-1 application (100 nM) for 30 s or 1 minute resulted in a large potentiation of capsaicin-activated currents through TRPV1 (Fig. 3A – C), like that in sensory neurons. Currents recorded during the first response to capsaicin directly after ET-1 application were increased 8.3 ± 1.5-fold (*n *= 10), those in response to the second capsaicin application, 2.5 minutes after ET-1 treatment, 10.7 ± 2.0-fold (*n *= 9). In addition, in most cells, ET-1 application resulted in the slow activation of a current, particularly in the outward direction, with the characteristics of TRPV1 (Fig. 3A, B). However, none of the cells displayed a rapid, transient current like that seen in DRGs, an indication that the ET-1-activated current inn DRGs may be mediated by a different channel.

In ET_A_R-expressing cells, application of ET-1 also potentiated currents activated by low pH or by heat (Fig. 3D – J). H^+^-activated TRPV1 currents were evoked by application of a solution of pH 5.1, a pH which produces a submaximal TRPV1 activation, preceded by the application of a solution of pH 6.8 to desensitize endogenous acid-sensing ion channels (ASICs; Fig. 3D) [36]. Application of ET-1 resulted in a 9.9 ± 1.3-fold (*n *= 12) potentiation of the first response to pH 5.1 after ET-1 application (Fig. 3D – F), and a significant potentiation of subsequent responses (Fig. 3D, F). Current responses of TRPV1 to heat were recorded upon raising the temperature from room temperature to 44°C (Fig. 3G). In controls, consecutive responses had similar amplitudes (Fig. 3G, *left*). ET-1 augmented the responses to the increase in temperature (2.5 ± 0.6-fold, *n *= 10, for the first response after ET-1, Fig. 3I) and, as illustrated for the experiment in Fig.3G, 3H, shifted the threshold for TRPV1 activation to lower temperatures (Fig. 3J).

**Figure 4:**
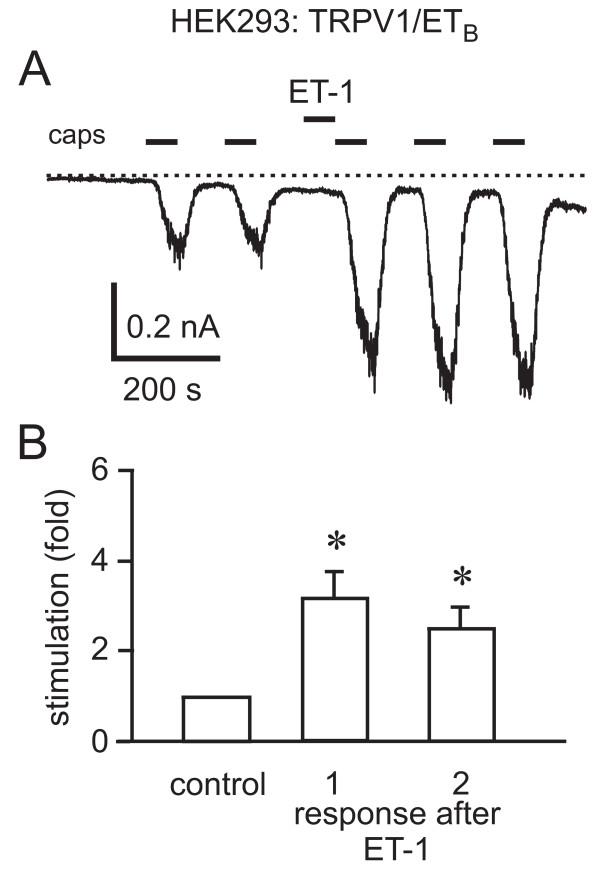
**ET-1 also potentiates capsaicin-activated currents in HEK293 cells cotransfected with TRPV1 and the ET_B_R**. A, Current responses to capsaicin (10 nM) application were potentiated by the application of ET-1 (100 nM). B, Bar graph summarizing the effects of ET-1 on normalized capsaicin responses in cells cotransfected with TRPV1-YFP and the ET_B_R. Shown are the first (*n *= 7, *p *= 0.0102) and second (*n *= 6, *p *= 0.0163) capsaicin response after ET-1 normalized to the capsaicin response before ET-1 application.

HEK293 cells transfected with the same amounts of TRPV1 and the ET_B_R showed a less prominent ET-1-induced potentiation of capsaicin-stimulated TRPV1 currents when compared to that elicited by the ET_A_R (Fig. 4). The first and second responses after ET-1 application were 2.9 ± 0.8-fold and 2.7 ± 0.5-fold (*n *= 5) that of the control, respectively.

### Role of protein kinases in the ET_A _receptor-mediated potentiation of TRPV1

Both ET receptor subtypes couple to G proteins from different families and each of the receptor types has its own distinct profile of G proteins that it can activate. The ET_A_R stimulates G proteins of the G_q_, G_s _and G_12/13 _families, the ET_B_R activates G proteins of the G_q_and G_i _families e.g. [37-40]. To investigate the signalling pathways activated and possibly involved in the modulation of TRPV1 in HEK293 cells, we studied cAMP and inositol phosphate formation in HEK cells stably expressing the ET_A_R or ET_B_R. ET-1 induced 22 ± 2.5- and 18 ± 2.0-fold increases in inositol phosphate formation *via *ET_A_Rs and ET_B_Rs, respectively (Table 1). For HEK cells expressing the ET_A_R, a 34 ± 10.6-fold increase in cAMP levels was noted. Cells expressing the ET_B_R did not show any ET-1-induced increase in cAMP.

**Table 1:** Synopsis of ET-1-mediated cAMP and inositol phosphate formation via ET_A _and ET_B _receptors

	HEK ET_A_R		HEK ET_B_R	
	-fold of control	EC_50 _[nM]	-fold of control	EC_50 _[nM]

formation of inositol phosphates	22.3 ± 2.5	4.5 ± 1.2	18 ± 2.0	7.3 ± 1.7
formation of cAMP	34 ± 10.6	13.3 ± 3.2	n. i.	n.a.

HEK293 cells stably expressing the ET_A_R or the ET_B_R were stimulated with increasing concentrations of ET-1 (up to 100 nM) in the presence of 10 mM LiCl (inositol phosphate assays) for 60 min or 1 mM IBMX (cAMP assays) for 30 min. The amount of formed inositol phosphates was determined by anion exchange chromatography and cAMP was determined by cAMP RIA as described in *Materials and Methods*. Values are means ± SD of at least three independent experiments performed in duplicate. Duplicates differed by less than 10 %. n.i.: no increase, n.a.: not applicable

**Figure 5:**
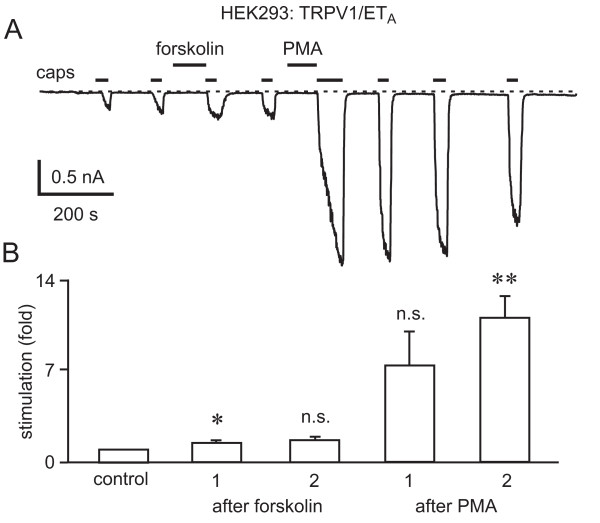
**Potentiatory effects of forskolin and PMA onTRPV1**. A, Adenylyl cyclase activation with forskolin (50 μM) resulted in a small increase in current responses to capsaicin (caps, 10 nM) whereas application of the PKC activator PMA (1 μM) resulted in a large potentiation of responses to capsaicin. B, Bar graph showing the control and the first two normalized responses to capsaicin after forskolin (forskolin 1: *n *= 5, *p *= 0.0271; forskolin 2: *n *= 4, *p *= 0.067) and PMA (PMA 1: *n *= 4, *p *= 0.0922; PMA 2: *n *= 4, *p *= 0.0088) application.

From the data presented above, stimulation of the G_q_-coupled ET_A_R in HEK293 cells could potentiate TRPV1 by a PLCβ-mediated breakdown of PIP_2 _or by the production of DAG and activation of PKC. Alternatively, TRPV1 potentiation could be mediated by the activation of PKA. We therefore studied the effects of PKC and adenylyl cyclase (AC)/PKA activation on capsaicin-mediated TRPV1 activation, and compared these with ET-1/ET_A_R-mediated effects on TRPV1 activity. Application of the phorbol ester, phorbol myristate acetate (1 μM), for 1 minute before the addition of capsaicin (10 nM) resulted in a clear and strong potentiation of the capsaicin responses that closely resembled the potentiation seen with ET-1 (Fig. 5). In contrast to activation of PKC by PMA, the activation of AC with forskolin (50 μM, Fig. 5) or treatment with the membrane-permeable cAMP analog, dibutyryl cAMP (dbcAMP; 5 mM), had comparatively weak effects or no effect on TRPV1 currents, respectively. After 1.5 minutes of treatment, forskolin increased currents 1.5 ± 0.1-fold (*n *= 4), but the effect after 4 minutes of forskolin treatment was not significant. Similarily, dbcAMP had no significant effect on current responses to capsaicin; they were 0.97 ± 0.11-fold (*n *= 4, *p *= 0.802) and 1.5 ± 0.5-fold (*n *= 4, *p *= 0.357) of the control, after 1.5 and 4 minutes of dbcAMP application, respectively.

**Figure 6:**
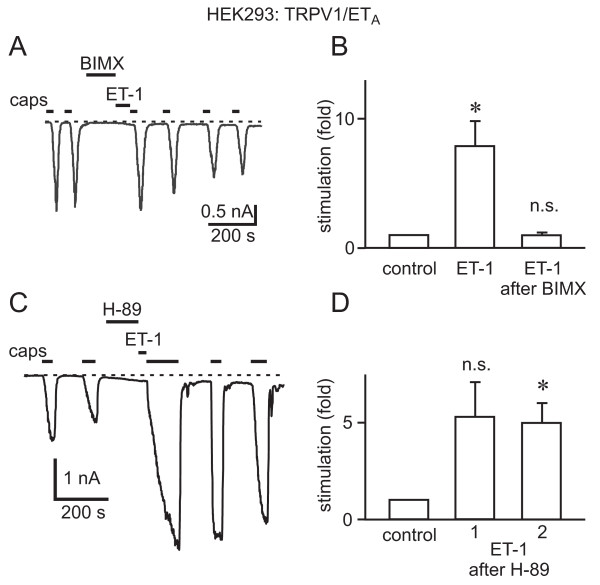
**Protein kinase C inhibitors, but not protein kinase A inhibitors prevent the potentiation of TRPV1 by ET-1**. A, Current trace from an experiment in which the PKC inhibitor BIM X (500 nM) was applied immediately prior to stimulation with ET-1 (100 nM) illustrating that BIM X treatment prevented potentiation by ET-1. B, Bar graph summarizing the effects of ET-1 on the first response to capsaicin after ET-1 in the absence (*n *= 6, *p *= 0.0156) and presence of BIM X (*n *= 7, *p *= 0.8964). C, Trace showing ET-1 potentiation of capsaicin-activated currents in the presence of the PKA inhibitor H-89. D, Bar graph summarizing the effects of ET-1 on the first (*n *= 4, *p *= 0.0953) and second (*n *= 4, *p *= 0.0275) capsaicin responses after treatment with H-89. Cells were cotransfected with TRPV1-YFP and the ET_A_R.

To analyse the role of PKC activation in ET-1-mediated potentiation of TRPV1, we tested the effect of the PKC inhibitor BIM X on the potentiation of capsaicin-induced TRPV1 currents in response to ET-1. Potentiation was completely prevented by a 2 minute incubation with BIM X (500 nM) prior to the ET-1 application (Fig. 6A). Control experiments performed in parallel on the same days with cells transfected at the same time showed strong potentiation (Fig. 6B). In contrast to the results after PKC inhibition, potentiation still occurred after treatment with the protein kinase inhibitor H89 (5 μM, *n *= 4), which at this concentration most strongly inhibits PKA, and was not significantly different from that without H89 (Fig. 6).

**Figure 7:**
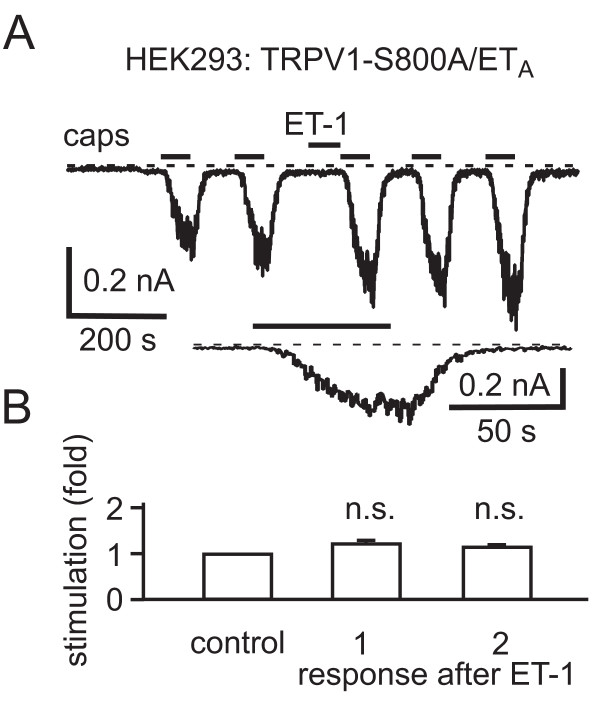
**ET-1 does not potentiate capsaicin responses from the PKC phosphorylation site mutant TRPV1-S800A**. A, top, Current trace showing responses to capsaicin (10 nM) before and after ET-1 (100 nM) application to a HEK293 cell cotransfected with TRPV1-S800A and the ET_A_R. Bottom, Current response to capsaicin after ET-1 in top trace on an expanded time scale. B, Bar graph summarizing the effects of ET-1 on the first (*n *= 4, *p *= 0.0735) and second (*n *= 4, *p *= 0.0921) capsaicin responses after ET-1 application recorded from TRPV1-S800A.

To confirm the pivotal role of PKC-mediated phosphorylation in the modulation of TRPV1 by ET-1 using an independent approach, we employed a mutant of TRPV1 which does not show PKC-mediated potentiation. The PKC phosphorylation sites involved in PKC-mediated enhancement of capsaicin-activated currents through TRPV1 have been localized to S502 and S800 [41,42]. Mutation of either site to alanine leads to a very strong reduction in sensitization of currents by PMA [41,42] and by mediators like ATP that act *via *the G_q/11_-coupled metabotropic P2Y_1 _receptor leading to an activation of PKC [41]. We therefore tested the effects of ET_A_R activation on the TRPV1 mutant TRPV1-S800A. The ET_A_R was coexpressed with YFP-tagged TRPV1-S800A in HEK293 cells. The mutant TRPV1-S800A differed from wild type TRPV1 in that currents tended to increase with consecutive responses, rather than decrease (compare the first and second response to capsaicin in Fig. 7A). In contrast to wild type TRPV1, no significant potentiation of capsaicin-activated currents was observed on application of ET-1 (Fig. 7). It is unclear whether the slight increase after ET-1 in Fig. 7A results from run-up of channel currents or from a very small potentiation that persisted in the mutant. The inability to observe an effect does not result from the briefer capsaicin application than that for the wild type because the application was continued until the current response was close to a plateau (Fig. 7A, lower trace). It is clear, however, that the strong stimulatory effect of ET_A_R activation is lost following mutation of the PKC phosphorylation site, consistent with sensitization resulting from PKC-mediated phosphorylation.

## Discussion

We show here that DRG neurons mainly express ET_A_Rs and that their expression partially overlaps with the expression of TRPV1. We also show that ET-1 potently modulates the functional activity of TRPV1 in a subpopulation of sensory neurons and in HEK293 cells co-expressing the ET_A_R and TRPV1. A significant modulation of TRPV1 activity was also found for HEK293 cells co-expressing TRPV1 and ET_B_Rs.

Even though the ET_A_R can stimulate pathways leading to both PKA and PKC activation, receptor-mediated potentiation of TRPV1 in HEK293 cells was predominantly mediated by PKC. Evidence for this is that ET-1 effects were completely inhibited by the PKC inhibitor BIM X, and prevented in the PKC phosphorylation site mutant TRPV1-S800A. There is also a strong similarity between the extent of potentiation by ET-1 and that elicited by the PKC-activating phorbol ester PMA. In contrast to the effects of PKC activation and inhibition, the effects of PKA activation by forskolin were comparatively weak, and dbcAMP had no significant effect. Potentiation by ET-1 also persisted in the presence of H-89, an inhibitor of PKA. In addition, some potentiation was observed with the ET_B_R which did not increase cAMP. These results indicate that, under the conditions used, i.e. in the absence of extracellular Ca^2+^, ET-1-mediated potentiation is unlikely to occur via G_s _and AC, nor to a great extent *via *PKC-mediated activation of AC. Because the cAMP/PKA pathway acts, at least partly, by decreasing Ca^2+^-dependent desensitization [32,43], we cannot rule out that this pathway provides an additional component of potentiation of TRPV1 by ET-1 at physiological Ca^2+ ^concentrations. Our work lends support to a recent study showing that PKC-ε is involved in the ET-1-mediated enhancement of capsaicin-induced Ca^2+ ^increases in sensory neurons, but which did not show that TRPV1 is the target for PKC-ε-mediated phosphorylation [19]. Our observation is also in line with the finding that S800 is crucially involved in PMA-mediated sensitization of TRPV1 via PKC-ε [44]. From our data, we cannot rule out that different pathways may be involved in the responses to ET-1 in sensory neurons, but there is a striking similarity between the effects in DRGs and in HEK293 cells. The evidence for an involvement of PKC in potentiation by ET-1 supports studies showing an involvement of PKC in potentiation of TRPV1 by bradykinin *via *B_2 _receptors [26,27,31], ATP *via *G_q_-coupled P_2_Y_1 _receptors [41,45], the chemokine CCL_3 _*via *CCR_1 _[46], 5-HT *via *5-HT_2 _receptors [47], and in some of the effects of prostaglandins *via *EP_1 _or IP receptors [48], but contrasts with that showing that the effects of bradykinin and NGF result from PLC-mediated release of TRPV1 from inhibition by PIP_2 _[28,49]. The increase of TRPV1 currents in response to PKC activation in sensory neurons most likely results from a phosphorylation-induced increase in the activity of channels at a given agonist concentration [50], but has also been attributed to an increased recruitment of intracellularly-stored vesicles carrying TRPV1 to the plasma membrane [51].

In DRG neurons, we observed two effects of ET-1; potentiation of responses to capsaicin and, in a smaller population of neurons, the activation of an inward current. These effects are very similar to those of bradykinin, which also activates an inward current, most likely a cation current, in some sensory neurons [26,52,53], and potentiates currents through TRPV1 [26,27,31]. It remains unclear whether the channel activated by bradykinin is TRPV1 because not all heat-sensitive neurons with temperature thresholds of 42°C, characteristic for TRPV1, show a current response to bradykinin [26]. On the other hand, high concentrations of bradykinin can shift the temperature threshold of TRPV1 sufficiently to produce a current at room temperature [27]. The inward current activated by ET-1 was not characterized directly, but it differed from TRPV1 currents in the absence of current noise for currents of comparable amplitudes, and there was no link between the amplitude of the ET-1-activated and capsaicin-activated currents. Other possible candidates for the ET-1-activated channel include other cation channels, like e.g. TRPA1, which has been shown to be activated by bradykinin [54]. It is also notable that in HEK293 cells co-transfected with TRPV1 and ET_A_Rs or ET_B_Rs, ET-1 did not induce a rapidly activating and inactivating inward current like that in DRG neurons, but did result in a small slow increase in an outwardly-rectifying current that resembled TRPV1. Thus, the molecular basis and nature of the fast inward current seen in DRG neurons in response to ET-1 remains to be clarified.

Our data at the cellular level support a role of potentiation of currents through TRPV1 in ET-1-induced excitation of nociceptors by increasing their sensitivity to algogenic stimuli. Furthermore, ET-1 could produce an initial transient excitation of some neurons by the activation of an inward current. Previous studies have postulated that part of the effects of ET-1 on nociception occur *via *modulation of TTX-resistant Na^+ ^channels [20] shifting their potential dependence of activation to more negative membrane potentials leading to enhanced excitability of the sensory neurons. Effects on Na^+ ^channels have been observed with the hyperalgesic modulators PGE_2_, 5-HT, epinephrine and adenosine (for reviews see [55,56]), and may be mediated by PKA or PKC activation [57]. Thus, the actions of ET-1 in sensory neurons involve contributions of Na^+ ^channels and TRPV1, and may be mediated by both PKA and PKC. By its concerted actions on TRPV1 and Na^+ ^channels, ET-1 could increase the depolarization of sensory endings in response to noxious stimuli and concomitantly reduce the threshold for activation of a population of Na^+ ^channels.

The effects of ET-1 on nociception are complex. Different experimental models have been used to study the role of ET-1 in nociception, and receptor subtype-specific agonists and antagonists to identify the ET receptor subtype mediating the effects. The pronociceptive actions of ET-1 have been reported to involve either ET_A_Rs [9,10,58-62] or ET_B_Rs [63,64], or both ET_A_Rs and ET_B_Rs [12,65,66]. Our results indicate that the ET_A_R is expressed in sensory neurons and could contribute to the algogenic effects of ET-1 by sensitizing TRPV1. This suggestion is supported by a recent study on mouse DRGs which showed that the Ca^2+ ^release in response to ET-1, and the potentiatory effect of ET-1 on capsaicin-induced Ca^2+ ^responses are mediated exclusively by ET_A_Rs [19]. Even though the ET_B_R is able to potentiate TRPV1 in HEK293 cells, the low level of ET_B_R expression in DRG neurons indicates that the algogenic effects of ET_B_R agonists [12,64] and the analgesic effects of ET_B_R antagonists [63-66] are more likely to result from indirect effects on the ET_B_R in Schwann and other glial cells where receptor expression is high [17]. It is difficult to extrapolate from data obtained on single isolated neurons to the situation *in vivo*, but our results could explain the excitatory effects of ET-1 injection on nociceptors and some of the amplification of responses to thermal and mechanical stimuli. The rapid inward current, if occurring in the periphery, could rapidly excite some ET receptor-expressing nociceptive neurons. Thereafter, the potentiation of TRPV1 could be responsible for the excitation, and for the hyperalgesia and allodynia seen after ET-1 application. Potentiation of TRPV1 with high ET-1 concentrations occurs rapidly and, although it decreases with time, by extrapolating our data (e.g. those in Fig. 2A) can probably persist for several tens of minutes. It is more difficult to explain the TRPV1-mediated prolongation of tactile allodynia in response to low ET-1 concentrations where a role of TRPV1 is only significant at times longer than 30 minutes after ET-1 application [67]. While the role of the ET_A_R in the action of ET-1 on nociception is relatively clear, the role of the ET_B_R is less well understood. The ET_B_R in glial cells and possibly in neurons is likely to have pronociceptive effects, whereas the ET_B_R in keratinocytes has been reported to mediate the analgesic effects of ET-1 and ET_B _agonists by inducing the release of β-endorphin [68].

## Conclusion

Our results show that ET-1 potentiates TRPV1 *via *a PKC-mediated effect and we suggest that this could be responsible for a major part of the pain-producing and hyperalgesic effects of ET-1 observed in previous studies.

## Methods

### Isolation and culture of DRG neurons

Dorsal root ganglia (DRG) were isolated as described recently [69]. In brief, DRG from lumbar segments L4 – L5 of male Wistar rats were isolated and placed in Minimal Essential Medium (MEM, Biochrom AG, Berlin, Germany) at 4°C. Thereafter, they were sequentially digested with collagenase (type II; 3 mg/ml) for 50 min at 37°C, and trypsin (type I; 0.25 mg/ml) for 10 min at 37°C. After careful mechanical dissociation, cells were washed twice and resuspended in fresh medium supplemented with 10% horse serum, 50 ng/ml NGF, 50 U/ml penicillin and 50 μg/ml streptomycin. Neurons were seeded on poly-l-lysine-coated glass coverslips in 6-well culture plates 4 – 36 h before the experiments. Neurons were kept at 37°C in an atmosphere of 5% CO_2_.

### Generation of plasmids encoding mutant TRPV1

To investigate the role of PKC-mediated phosphorylation of TRPV1, a plasmid was generated, which encodes a mutant TRPV1 in which serine 800 is replaced by alanine (TRPV1-S800A). Site-directed mutagenesis was performed with the QuickChange mutagenesis kit (Stratagene, La Jolla, CA), using a plasmid encoding a rat TRPV1-YFP fusion protein [70] and appropriate sense and antisense oligonucleotides spanning the mutated triplet and 15 flanking nucleotides. The construct was verified by DNA sequencing.

### HEK culture

HEK293 cells were cultured in MEM-Earle medium (Biochrom), supplemented with 10% (v/v) fetal calf serum (Gibco/Invitrogen, Karlsruhe, Germany) and 100 U/ml penicillin and 100 μg/ml streptomycin. Cells were plated onto glass cover slips 24 – 48 h prior to transfection. The cells were transiently transfected with 1 μg of the plasmid pTRPV1-YFP [70] and 2 μg of the plasmid pET_A_-myc DNA [71] or the plasmid containing the FLAG-tagged ET_B _DNA [71] using 6 μl of FuGENE 6 Transfection Reagent (Roche Diagnostics, Mannheim, Germany) in 94 μl of OptiMEM medium (Gibco/Invitrogen) per 85 mm dish. In some experiments, cells were transfected 1 – 2 days after plating into 35 mm dishes using 9 μl TransIT (Mirus, Madison, WI) in serum-free MEM-Earle medium, subsequently trypsinized and plated onto glass coverslips 2 days after transfection. No differences were observed between results obtained with the two methods. Electrophysiological experiments were performed 24 – 72 h after transfection.

### Immunofluorescence analysis

Immunofluorescence analysis was performed as described recently [69]. Freshly isolated DRG were embedded in Tissue-Tek compound (OCT, Miles Inc., Elkhart, USA) and frozen. Consecutive sections (9 μm) were prepared with a cryostat and mounted onto gelatin-coated slides. To prevent non-specific binding, the sections were incubated for 60 min in PBS containing 0.3% Triton X-100, 1% BSA, 4% goat serum, and 4% horse serum (block solution). The sections were then incubated overnight at 4°C with a guinea pig polyclonal antibody against TRPV1 (1:1,000; Chemicon, CA, USA) or S100 Antigen (1:200; Abcam, Cambridge, UK) in combination with a peptide-derived rabbit polyclonal antibody directed against the C terminus of the ET_A_R (1:100) (Plant et al., 2006) or against the N terminus of the ET_B_R (1:100) [71]. The tissue sections were washed with PBS and then incubated with Texas red-conjugated goat anti-rabbit antibody and FITC-conjugated donkey anti-guinea pig antibody. Thereafter, sections were washed with PBS, mounted in vectashield (Vector Laboratories, Burlingame, CA, USA) and viewed with a Zeiss 510 laser scanning microscope (Zeiss, Oberkochen, Germany).

### cAMP and inositol phosphate measurements

Determination of inositol phosphates and of cAMP was performed as described previously [72]. In brief, HEK293 cells stably expressing ET_A_Rs or ET_B_Rs were seeded onto 24-well plates (100,000 cells/well). For inositol phosphate determination, cells were incubated the following day with 74 kBq/ml myo- [2-^3^H]inositol (specific activity 370 – 740 GBq/mmol; Amersham Biosciences) for 20 h at 37°C. Cells were then washed with DMEM, 10 mM HEPES, 0.5% BSA, 10 mM LiCl, and finally stimulated with buffer or increasing concentrations of ET-1 (10 pM to 100 nM) for 60 min at 37°C. Cells were then lysed with 0.1 M NaOH, and inositol phosphates isolated from cleared supernatants by anion exchange chromatography.

For the determination of cAMP, cells were washed with 1 ml of stimulation medium (DMEM without fetal calf serum, supplemented with 10 mM HEPES, 0.5% BSA, 0.25 mM 3-isobutyl-1-methylxanthine) and incubated for 30 min at 37°C with buffer or increasing concentrations of ET-1 (10 pM to 100 nM). Cells were extracted with 750 μl of 0.1% trifluoroacetic acid, 0.005% Triton X-100 for 30 min at 4°C. After acetylation of the samples, the cAMP content was determined using ^125^I-cAMP-tyrosylmethylester (10,000 cpm, specific activity 81.4 TBq/mM, Biotrend, FRG) and polyclonal rabbit anti-cAMP-antibody (final dilution 1:160,000). After an overnight incubation at 4°C, the antibody-bound fraction was precipitated, and the radioactivity of the precipitate was determined in a β-counter.

### ^125^I-ET-1 binding analysis

Binding analysis was performed as described [73,74]. In brief, membranes (5 μg) of DRGs were incubated in a final volume of 200 μl Tris/BAME buffer with increasing concentrations of ^125^I-ET-1 (18 to 1000 pM) in the absence or presence of unlabelled ET-1 (1 μM) (to detect ET_A_Rs and ET_B_Rs) for 2 hours at 25°C. The ratio of ET_A _and ET_B_R expression was determined by ^125^I-ET-1 saturation binding analysis using ET-1 (1 μM, total binding), IRL1620 (1 μM, ET_B_R-specific binding) or BQ123 (10 μM, ET_A_R-specific binding) as competing ligands. The samples were then transferred onto GF/C filters (Whatman International Ltd., Maidstone, UK), and washed twice with PBS using a Brandel cell harvester. Radioactivity was determined in a γ-counter. Data were analyzed with RadLig Software 4.0 (Cambridge, UK).

### Patch clamp recordings

Recordings of whole cell currents from single cells were made with an EPC-7 or EPC-10 amplifier using Pulse software (HEKA, Lambrecht, Germany) as described previously [75]. Experiments were performed using the standard whole cell mode of the patch clamp technique. Cells were held at a potential of -60 mV and the current recorded using XChart (HEKA). In HEK293 cells, ramps from -100 to +100 mV with a duration of 400 ms were applied at a frequency of 0.2 Hz. Ramp data were acquired at a frequency of 4 kHz after filtering at 1 kHz.

The standard pipette solution contained 100 mM CH_3_O_3_SCs (cesium methane sulfonate), 25 mM CsCl, 3 mM MgCl_2_, 2 mM Na_2_ATP, 3.62 mM CaCl_2_, 10 mM EGTA, 30 mM HEPES (pH 7.2 with CsOH). Pipette tips were filled with the same solution without ATP to avoid the activation of purinergic receptors prior to seal formation. The standard extracellular solution contained: 140 mM NaCl, 5 mM CsCl, 2 mM CaCl_2_, 1 mM MgCl_2_, 10 mM glucose, 10 mM HEPES (pH 7.4 with NaOH). For Ca^2+^-free solutions, Ca^2+ ^was omitted and 0.5 mM EGTA added. Experiments were performed at room temperature 20 – 25°C. Solutions were applied by bath perfusion. In most cases, with the exception of Fig. 2B, which was not used for quantification of the response, capsaicin was applied until currents approached a steady state value. In experiments on the effects of heat, bath temperature was measured using a thermistor placed close to the cell studied and recorded using XChart. The test solution was preheated to appropriate temperatures using a water jacket connected to a water bath.

## Competing interests

The author(s) declare that they have no competing interests.

## Authors' contributions

TP conceived and designed the study, performed the electrophysiological experiments, analyzed the data and drafted the manuscript. CZ isolated the DRGs for immunohistochemical work and binding experiments, established primary cultures and perfomed part of the cAMP and inositol phosphate experiments. FK performed electrophysiological experiments and analyzed the data. SSM performed the immunohistochemistry. JE conducted binding experiments with membrane preparations of DRGs. MS provided rTRPV1-YFP and generated the TRPV1-S800A mutant. JF conducted part of the cAMP and inositol phosphate experiments, and performed analysis of cAMP and inositol phosphates from cell extracts. CS supervised the part of the study on DRGs. AO conceived and designed the study, performed laser scanning microscopy, supervised binding experiments and respective analysis, and drafted the manuscript.

All authors read and approved the manuscript.
